# Supplementary material for: “I found out about Zika virus after she was born.” Women’s experiences of risk communication during the Zika virus epidemic in Brazil, Colombia, and Puerto Rico
Source: PLOS Glob Public Health. 2024 Jun 12;4(6):e0002808. doi: 10.1371/journal.pgph.0002808 (PMC11168637; doi:10.1371/journal.pgph.0002808)
Supplement: S1 Table — (DOCX) [file pgph.0002808.s001.docx]

| **Variable** | **Puerto Rico** | **Colombia: Bucaramanga** | **Colombia: Barranquilla** | **Colombia: Neiva** | **Brazil: Pernambuco** | **Brazil: Rio de Janeiro** | **Brazil: Paraíba** |
| --- | --- | --- | --- | --- | --- | --- | --- |
| **Source population** |  |  |  |  |  |  |  |
| **pregnant during ZIKV epidemic; no ZIKV outcome** | Women receiving services through maternal units of hospitals in Puerto Rico, clinics, government services through the Dept. of Health's Visitas al Hogar, WIC and Familias Saludables), community based organizations (SePARE, ASI, Partneras del Sur), research service program (Comienzo Saludable). | Women who were pregnant during the Zika outbreak and enrolled in a cohort study (ZikAlliance and ZEN) linked to their regular and high-risk antenatal care, and receiving regular prenatal care through the antenatal control care service of the hospital and health care centers. Women in this group will have received a positive test for or been a suspected case of Zika but their pregnancy did not have a Zika-related outcome. | Women who were pregnant during the Zika outbreak and had Zika related symptoms during gestation but did not have a Zika-related outcome. These women were enrolled in the cohort: “Comprehensive follow up of infants and children with prenatal Zika virus exposure” | Women who were pregnant during the Zika outbreak and had Zika related symptoms during gestation but did not have a Zika-related outcome. These women were enrolled in the cohort: “Comprehensive follow up of infants and children with prenatal Zika virus exposure” | UMA (União de Mães de Anjos) women's group in Pernambuco. Women pregnant during the ZIKA outbreak will be recruited through mothers’ associations. The site investigators will contact association coordinators (gatekeepers) and will ask their help in identifying and inviting women to participate. The association coordinators will introduce women to the study team so they can explain the study in detail and ask them if they would like to participate and/or be willing to tell other women about the study and pass along the contact information. The study team will also ask women that do and do not agree to participate if they know of anyone who might like to participate, and if they would be willing to give the contact information of the study team to that person so she could contact them to learn about the study (snowball recruitment). | LOTUS Association women's group in Rio de Janeiro. Women pregnant during the ZIKA outbreak will be recruited through mothers’ associations. The site investigators will contact association coordinators (gatekeepers) and will ask them to help by identifying eligible women and inviting them to talk to the study team about participating in the study. The mothers’ association coordinators will introduce women to the study team so they can explain the study in detail and ask them if they would like to participate. | Professor Joaquim Amorim Neto Research Institute (IPESQ) has provided specialist care, conducted research, and followed women with Zika infection since 2015. IPESQ is following over 150 mothers and their children, and has direct contact with them through its treatment programs and regional mother’s associations. The site investigators will work with the \IPESQ coordinators and will ask their help in identifying and inviting women to participate. The IPESQ coordinators will help to identify and contact mothers to research team via WhatsApp message or a phone call, so the research team can explain the study in detail and ask them if they would like to participate. |
| **pregnant during ZIKV epidemic; ZIKV outcome** | Women receiving services through maternal units of hospitals in Puerto Rico, clinics, government services through the Dept. of Health's Visitas al Hogar, WIC and Familias Saludables), community based organizations (SePARE, ASI, Partneras del Sur), research service program (Comienzo Saludable). | Women who were pregnant during the Zika outbreak and enrolled in the cohort study (Zen) as part of their regular and high-risk antenatal care, and receiving regular prenatal care through the antenatal control care service of the hospital and health care centers. This group will have been tested positive or been a suspected case of Zika but whose pregnancy did not have a Zika-related outcome. | Women who were pregnant during the Zika outbreak and had Zika related symptoms during gestation. Some of this group could have been tested for Zika infection, and had either a positive or negative test and had a Zika-related outcome. These women were enrolled in the cohort: “Comprehensive follow up of infants and children with prenatal Zika virus exposure” | Women who were pregnant during the Zika outbreak and had Zika related symptoms during gestation. Some of this group could have been tested for Zika infection, and had either a positive or negative test and had a Zika-related outcome. These women were enrolled in the cohort: “Comprehensive follow up of infants and children with prenatal Zika virus exposure” | UMA (União de Mães de Anjos) women's group in headquartered in Recife but that includes members from throughout Pernambuco. Women pregnant during the ZIKA outbreak will be recruited through the UMA association. The site investigators will contact association coordinators (gatekeepers) and will ask their help in identifying and inviting women to participate. The UMA association coordinators will introduce women to the study team so they can explain the study in detail and ask them if they would like to participate and/or be willing to tell other women about the study and pass along the contact information. | LOTUS Association women's group in Rio de Janeiro. Women pregnant during the ZIKA outbreak will be recruited through the LOTUS mothers’ association. The site investigators will contact association coordinators (gatekeepers) and will ask them to help by identifying eligible women and inviting them to talk to the study team about participating in the study. The mothers’ association coordinators will introduce women to the study team so they can explain the study in detail and ask them if they would like to participate. and/or be willing to tell other women about the study and pass along the contact information. | Professor Joaquim Amorim Neto Research Institute (IPESQ) has provided specialist care, conducted research, and followed women with Zika infection since 2015. IPESQ is following over 150 mothers and their children, and has direct contact with them through its treatment programs and regional mother’s associations. The site investigators will work with the \IPESQ coordinators and will ask their help in identifying and inviting women to participate. The IPESQ coordinators will help to identify and contact mothers to research team via WhatsApp message or a phone call, so the research team can explain the study in detail and ask them if they would like to participate. and/or be willing to tell other women about the study and pass along the contact information. |
| **Name of cohort study and cohort study PI women who were pregnant during the ZIKV epidemic were recruited from an ongoing or previous cohort of pregnant women** | N/A - recruitment will not happen through cohorts. | Started during Zika Outbreak (2016-now) 1. “Evaluación de riesgo de alteraciones del sistema nervioso fetal y neurodesarrollo en hijos de gestantes con infección con el virus Zika” Iniciativa ZEN/ Universidad Industrial de Santader Investigador Principal: Dr. Carlos Becerra. Co-investigador Dr Luis Villar.   Started Post Zika 2017:  2. “Cohorte de mujeres embarazadas para evaluar los riesgos de malformaciones congénitas y otros desenlaces adversos del embarazo después de infectarse con el virus Zika durante el embarazo - ZIKAlliance Cohort”. PI Thomas Jaenisch Local Cohorts PI: Dr. Luis Villar. | Started during the Zika Outbreak in March 2017 and is on-going.    Study name: “Comprehensive follow-up of infants and children with prenatal Zika virus exposure.”    PI: Marcela Mercado  National Institute of Health, Colombia | Started during the Zika Outbreak in March 2017 and is on-going.    Study name: “Comprehensive follow-up of infants and children with prenatal Zika virus exposure.”    PI: Marcela Mercado  National Institute of Health, Colombia | N/A - recruitment will not happen through cohorts | N/A - recruitment will not happen through cohorts | Started during Zika Outbreak (2015-now)  Study name: “Mulheres e crianças com história sugestiva de arboviroses durante a gestação”. The cohort is conducted by IPESQ and is coordinated by the PI.  PI: Dra. Adriana Melo. |
| **Sampling criteria** |  |  |  |  |  |  |  |
| **pregnant during ZIKV epidemic; no ZIKV outcome** | Women pregnant during the Zika epidemic and who did not have an identifiable Zika- related, adverse outcome to their pregnancy. | Women over the age of 18 who were pregnant during the Zika epidemic and who did not have an identifiable Zika outcome to their pregnancy. We will recruit for women of different parities from among the cohort participants (list sampling). | Women over the age of 18 who were pregnant during the Zika epidemic, were identified through the National surveillance system as having Zika related symptoms, with or without positive test results for Zika, and who did not have an identifiable Zika outcome to their pregnancy. We will recruit for women of different parities from among the cohort participants (list sampling). | Women over the age of 18 who were pregnant during the Zika epidemic, were identified through the National surveillance system as having Zika related symptoms, with or without positive test results for Zika, and who did not have an identifiable Zika outcome to their pregnancy. We will recruit for women of different parities from among the cohort participants (list sampling). | Women over the age of 18 who were pregnant during the Zika outbreak and did not have identifiable adverse fetal outcome at birth. We will include women who had different parities when they were pregnant during the 2015-2016 ZIKV outbreak. They will be recruited from the UMA women's group in Pernambuco. | Women over the age of 18 who were pregnant during the Zika outbreak and did not have identifiable adverse fetal outcome at birth. We will include women who had different parities when they were pregnant during the 2015-2016 ZIKV outbreak. They will be recruited from the Lotus Association Women's Group in Rio de Janeiro. | Women over the age of 18 who were pregnant during the Zika outbreak and did not have identifiable adverse fetal outcome at birth. We will include women who had different parities when they were pregnant during the 2015-2016 ZIKV outbreak. They will be recruited from IPESQ (Research Institute) in Paraiba. |
| **pregnant during ZIKV epidemic; ZIKV outcome** | Women pregnant during the Zika epidemic and who had a Zika-related, adverse outcome to their pregnancy. This includes women whose infant was born with CZS as well as early term birth and late-term miscarriage or still birth due to Zika infection. We will seek women with different outcomes, and of different parity if possible. | Women over the age of 18 who were pregnant during the Zika outbreak and had an adverse fetal outcome at birth. We will recruit for women of different parities and outcomes from among the cohort participants (list sampling). | Women over the age of 18 who were pregnant during the Zika epidemic, were identified through the National surveillance system as having Zika related symptoms, with or without positive test results for Zika, and who had an identifiable adverse fetal Zika outcome to their pregnancy. We will recruit for women of different parities from among the cohort participants (list sampling). | Women over the age of 18 who were pregnant during the Zika epidemic, were identified through the National surveillance system as having Zika related symptoms, with or without positive test results for Zika, and who had an identifiable adverse fetal Zika outcome to their pregnancy. We will recruit for women of different parities from among the cohort participants (list sampling). | Women over the age of 18 who were pregnant during the Zika outbreak and had an adverse fetal outcome at birth. We will include women who had different parities when they were pregnant during the 2015-2016 ZIKV outbreak. They will be recruited from the UMA women's group in Pernambuco. | Women over the age of 18 who were pregnant during the Zika outbreak and had an adverse fetal outcome at birth. We will include women who had different parities when they were pregnant during the 2015-2016 ZIKV outbreak. They will be recruited from Lotus Association Women's Group in Rio de Janeiro. | Women over the age of 18 who were pregnant during the Zika outbreak and had identifiable adverse fetal outcome at birth. We will include women who had different parities when they were pregnant during the 2015-2016 ZIKV outbreak. They will be recruited from IPESQ (Research Institute) in Paraiba. |
| **Participant recruitment process** |  |  |  |  |  |  |  |
| **Pregnant during ZIKV epidemic; no ZIKV outcome** | Staff and collaborators of the study, who are linked to past or on-going studies in PR and/or care service of local hospitals and health care centers, will contact clinicians, nursing staff and community-based organizations about the study. Collaborators at these institutions will invite women to participate in the qualitative study, and will provide the contact information of the study team. Women interested in participating who contact the study team and who agree to participate in the study and provide informed consent will do so by phone, or a team member will visit their homes if permitted or requested by the potential study participant. All women invited to participate, will read and/or review the consent form with a study team member and sign the consent form before any further contact. Study team members may approach women in the waiting areas of antenatal clinics and invite them to participate. If they express an interest in participating, they will be given the option of going to a private room to read through and sign the consent from at that time, or contacting the study team to set up a time and location of their choosing for the consent process. Women who agree to participate and consent may be asked to refer other women that they know who are currently pregnant, or were pregnant during the Zika epidemic to contact the study team to participate (snowball recruitment). Efforts will be made to assign women who know each other to different discussion groups. There is no clear permission in the Cohort ICF that allows for contacting women for other studies, so recruitment will be undertaken through partner organizations. | The nurse/social worker of the study that is linked to the cohort studies and to the antenatal control care service of the hospital and Health care centers will invite the women to participate in the qualitative study by phone or by visiting their homes if permitted by the potential study participant. This will take place within the month prior to the date of the focus group. A follow-up call will be made to remind the participant to attend the appointment. In the cohort studies’ consent forms, it is not explicit that the study will contact women for other purposes nor do they give explicit permission to be contacted to participate in other related studies. For this reason, we requested permission from the ethics committee t to re-contact these mothers for the ZIKV Qualitative study. All women will be invited to participate, and will read and sign the new consent form before any other contact or procedure | The site investigators who are linked to the cohort study will invite women to participate in the qualitative study by phone. Women interested in participating will be scheduled for a follow-up call to review the informed consent form and provide their verbal consent. This will be done within the month prior to the date of the focus group and then a call will be made to remind them to attend the discussion. | The site investigators who are linked to the cohort study will invite women to participate in the qualitative study by phone. Women interested in participating will be scheduled for a follow-up call to review the informed consent form and provide their verbal consent. This will be done within the month prior to the date of the focus group and then a call will be made to remind them to attend the discussion. | UMA mother's group in Pernambuco. UMA includes most of the mothers who experienced a ZIKV-related pregnancy outcome (CZS, microcephaly, etc.) in Pernambuco. Dr. Pimentel has worked regularly with UMA for other research studies related to the social impacts of ZIKV infection during pregnancy. S sampling.  The site investigators will contact the Coordinator of UMA association in Recife to ask her help in inviting women to participate. The coordinator of UMA will introduce women to the study team via WhatsApp message or a phone call, so the team can explain the study in detail and ask them if they would like to participate. The site investigator will send the informed consent form (ICF) and schedule the in-depth interview with women that decide to participate and are eligible. The ICF will be sent as an audio message and as document, either by e-mail or WhatsApp, according to the interviewee's preference.  The study team will also ask women that do and do not agree to participate if they know of anyone who might like to participate, and if they would be willing to give the contact information of the study team to that person so she could contact them to learn about the study (snowball recruitment). | The site investigators will contact the Coordinator of LOTUS association in Rio de Janeiro to ask her help in inviting women to participate. The coordinator of LOTUS will introduce women to the study team via WhatsApp message or a phone call, so the team can explain the study in detail and ask them if they would like to participate. The site investigator will send the informed consent form (ICF) and schedule the in-depth interview with women that decide to participate and are eligible. The ICF will be sent as an audio message and as document, either by e-mail or WhatsApp, according to the interviewee's preference.  The study team will also ask women that do and do not agree to participate if they know of anyone who might like to participate, and if they would be willing to give the contact information of the study team to that person so she could contact them to learn about the study (snowball recruitment). | The site investigators are part of the IPESQ (Research Institute) team. The investigators team will contact mothers via WhatsApp message or a phone call, and a team member will explain the study in detail and ask them if they would like to participate. The site investigator will send the informed consent form (ICF) and schedule the in-depth interview with the women that decide to participate and are eligible. The ICF will be sent as an audio message and as a document, either by e-mail or WhatsApp, according to the interviewee's preference. The study team will also ask women that do and do not agree to participate if they know of anyone who might like to participate, and if they would be willing to give the contact information of the study team to that person so she could contact them to learn about the study (snowball and list recruitment). |
| **Pregnant during ZIKV epidemic; ZIKV outcome** | Staff and collaborators of the study, who are linked to past or on-going studies in PR and/or care service of local hospitals and health care centers, will contact clinicians, nursing staff and community-based organizations about the study. Collaborators at these institutions will invite women to participate in the qualitative study, and will provide the contact information of the study team. Women interested in participating who contact the study team and who agree to participate in the study and provide informed consent will do so by phone, or a team member will visit their homes if permitted or requested by the potential study participant. All women invited to participate, will read and/or review the consent form with a study team member and sign the consent form before any further contact. Study team members may approach women in the waiting areas of antenatal clinics and invite them to participate. If they express an interest in participating, they will be given the option of going to a private room to read through and sign the consent from at that time, or contacting the study team to set up a time and location of their choosing for the consent process. Women who agree to participate and consent may be asked to refer other women that they know who are currently pregnant, or were pregnant during the Zika epidemic to contact the study team to participate (snowball recruitment). Efforts will be made to assign women who know each other to different discussion groups. There is no clear permission in the Cohort ICF that allows for contacting women for other studies, so recruitment will be undertaken through partner organizations. | The nurse/social worker of the study that is linked to the cohort studies and to the antenatal control care service of the hospital and Health care centers will invite the women to participate in the qualitative study by phone or by visiting their homes if permitted by the potential study participant. This will take place within the month prior to the date of the focus group. A follow-up call will be made to remind the participant to attend the discussion. In the cohort studies’ consent forms, it is not explicit that the study will contact women for other purposes nor do they give explicit permission to be contacted to participate in other related studies. For this reason, we requested permission from the ethics committee t to re-contact these mothers for the ZIKV Qualitative study. All women will be invited to participate, and will read and sign the new consent form before any other contact or procedure. | The site investigators who are linked to the cohort study will invite women to participate in the qualitative study by phone. This will be done within the month prior to the date of the focus group and then a call will be made to remind the participant to attend the discussion. |  | The site investigators will contact the Coordinator ofUMA, the local mother’s association in Recife, and will ask her help to invite women to participate. The coordinator of UMA will introduce women to the study team via WhatsApp message or a phone call, so the team can explain the study in detail and ask them if they would like to participate.  The site investigator will send the informed consent form (ICF) and schedule the in-depth interview with the women that decide to participate and are eligible. The ICF will be sent as an audio message and as document, either by e-mail or WhatsApp, according to the interviewee's preference.  l The study team will also ask women that do and do not agree to participate if they know of anyone who might like to participate, and if they would be willing to give the contact information of the study team to that person so she could contact them to learn about the study (snowball recruitment). | The site investigators will contact the Coordinator of LOTUS association in Rio de Janeiro and will ask her help to invite women to participate. The coordinator of LOTUS will introduce women to the study team via WhatsApp message or a phone call, so the team can explain the study in detail and ask them if they would like to participate.  The site investigator will send the informed consent form (ICF) and schedule the in-depth interview with the women that decide to participate and are eligible. The ICF will be sent as an audio message and as document, either by e-mail or WhatsApp, according to the interviewee's preference.  l The study team will also ask women that do and do not agree to participate if they know of anyone who might like to participate, and if they would be willing to give the contact information of the study team to that person so she could contact them to learn about the study (snowball recruitment). | The site investigators are part of IPESQ (Research Institute) team. The investigators team will contact mothers by WhatsApp message or a phone call, so the team will explain the study in detail and ask them if they would like to participate. The site investigator will send the informed consent form (ICF) and schedule the in-depth interview with the women that decide to participate and are eligible. The ICF will be sent as an audio message and as document, either by e-mail or WhatsApp, according to the interviewee's preference. The study team will also ask women that do and do not agree to participate if they know of anyone who might like to participate, and if they would be willing to give the contact information of the study team to that person so she could contact them to learn about the study (snowball recruitment). |
| **Informed consent process** |  |  |  |  |  |  |  |
| **In-person interviews** | Potential participants who are interested in participating will undergo screening for eligibility and invited to participate. Study personnel will brief the women about the objectives and procedures of this study. These participants will receive a copy of the research Consent Form for them to study the purpose of the study, procedures, risk and benefits of the study, and reviewed with a member of the study team in a language of their choice (Spanish or English). Written informed consent (in English or Spanish, according to individual preferences) will be obtained from each subject prior to voluntary participation. The study will be promoted at clinics by study staff in waiting areas and by clinic staff and community gate keepers during clinic or center visits. Prospective participants will be consented by individuals not connected to them to ensure they do not feel coerced to participate. | The informed consent process will be carried out at the hospital or at the potential participant’s home. One of the co-researchers supported by the study's social worker will obtain the consent of the potential participants. The social worker (she is a women) has previously contact with these potential participants as part of the antenatal control and/or participation in cohort studies, so they already know her, it is a relationship of mutual trust and respect. | The informed consent process will be carried out at the research site prior to the FGD or in-depth interview (if post-COVID in-person FGDs will be conducted). This informed consent will be taken by the study´s public health technician, who is acquainted with the process and with the cohort participants. | The informed consent process will be carried out at the research site prior to the FGD or in-depth interview (if post-COVID in-person FGDs will be conducted). This informed consent will be taken by the study´s public health technician, who is acquainted with the process and with the cohort participants. | The site investigator will send the informed consent form (ICF) and schedule the focus group discussion or in-depth interview with the women that decide to participate and are eligible. The ICF will be sent as an audio message and as document, either by e-mail or WhatsApp, according to the interviewee's preference. The oral informed consent will be carried out at the beginning of the scheduled interview, prior to the IDI. The investigators will talk with them about all the issues related to their participation in the study, will ask them if they understand it and if they want to participate.  The investigators will talk about all the issues related on women's participation in the study, will ask them if they understand it and if they want to participate. | The site investigator will send the informed consent form (ICF) and schedule the focus group discussion or in-depth interview with the women that decide to participate and are eligible. The ICF will be sent as an audio message and as document, either by e-mail or WhatsApp, according to the interviewee's preference. The oral informed consent will be carried out at the beginning of the scheduled interview, prior to the IDI. The investigators will talk with them about all the issues related to their participation in the study, will ask them if they understand it and if they want to participate.  The investigators will talk about all the issues related on women's participation in the study, will ask them if they understand it and if they want to participate. | The site investigator will send the informed consent form (ICF) and schedule the focus group discussion or in-depth interview with the women that decide to participate and are eligible. The ICF will be sent as an audio message and as document, either by e-mail or WhatsApp, according to the interviewee's preference. The oral informed consent will be carried out at the beginning of the scheduled interview, prior to the IDI. The investigators will talk about all the issues related to women's participation in the study, will ask them if they understand it and if they want to participate. The investigators will talk about all the issues related on women's participation in the study, will ask them if they understand it and if they want to participate. |
| **Informed consent for remote interviews, necessitated by COVID-19 pandemic** | Potential participants who are interested in participating will undergo screening for eligibility and invited to participate via phone. If potential participants are interested in learning more, study personnel will brief the women about the objectives and procedures of this study. These participants will receive a copy of the research Consent Form for them to study the purpose of the study in Spanish or English that includes procedures, possible risks and benefits of the study. She will have the opportunity to review it with a member of the study team. Written informed consent (in English or Spanish, according to individual preferences) or oral consent, will be obtained from each subject prior to voluntary participation. Participants will be consented over the phone or via an online form. If online, they will be able to access a link with the consent and click a box to digitally sign. If taken over the phone, consent will be audiorecorded, The study will be promoted via study staff and with the assistance of clinic staff and community gate keepers via phone during check-ups and social media. Prospective participants will be consented by individuals not connected to them to ensure they do not feel coerced to participate. | Potential participants who are interested in participating will undergo screening for eligibility and be invited to participate by phone (by a social worker or a nurse). Second, if they are interested, study personnel (PI and co-PI) will call them to brief the women about the objectives and procedures of this study. This process will be documented by audio recording consent (each subject who agrees to participate will be asked to state that they understand the study and questions have been answered to their satisfaction, they are participating freely and voluntarily and give their permission to be recorded). After that, the study personnel will conduct the social demographic questionnaire and the in-depth interview. | First, potential participants who are interested in participating will undergo screening for eligibility and invited to participate by phone (by a public health technician affiliated with the study). Second, study personnel (PI and co-PI) will call them to brief the women about the objectives and procedures of this study. This process will be documented by audio recording consent (each subject who agrees to participate will be asked to state that they understand the study and questions have been answered to their satisfaction, they are participating freely and voluntarily and give their permission to be recorded). After that, the study personnel will conduct the social demographic questionnaire and the in-depth interview. | First, potential participants who are interested in participating will undergo screening for eligibility and invited to participate by phone (by a public health technician affiliated with the study). Second, study personnel (PI and co-PI) will also by phone brief the women about the objectives and procedures of this study. This process will be documented by audio recording consent (each subject who agrees to participate will be asked to state that they understand the study and questions have been answered to their satisfaction, they are participating freely and voluntarily and give their permission to be recorded). After that, the study personnel will conduct the social demographic questionnaire and the in-depth interview. | The informed consent process will be carried out by contacting potential participants by phone. The investigators will talk about all the issues related toon women's participation in the study, will ask them if they understand it and if they want to participate.  The site investigator will send the informed consent form (ICF) and schedule the in-depth interview with the women that decide to participate and are eligible. The ICF will be sent as an audio message and as a document, either by e-mail or WhatsApp, according to the interviewee's preference. The oral informed consent will be carried out at the beginning of the scheduled interview, prior to the IDI. The investigators will talk about all the issues related to women's participation in the study, will ask them if they understand it and if they still want to participate. Their verbal consent will be recorded. | The informed consent process will be carried out at the same day of the focus group discussion, prior to the FGD. The investigators will talk about all the issues related on women's participation in the study, will ask them if they understand it and if they want to participate.  The site investigator will send the informed consent form (ICF) and schedule the in-depth interview with the women that decide to participate and are eligible. The ICF will be sent as an audio message and as a document, either by e-mail or WhatsApp, according to the interviewee's preference. The oral informed consent will be carried out at the beginning of the scheduled interview, prior to the IDI. The investigators will talk about all the issues related to women's participation in the study, will ask them if they understand it and if they still want to participate. Their verbal consent will be recorded. | The site investigator will send the informed consent form (ICF) and schedule the in-depth interview with the women that decide to participate and are eligible. The ICF will be sent as an audio message and as document, either by e-mail or WhatsApp, according to the interviewee's preference. The oral informed consent will be carried out at the beginning of the scheduled interview, prior to the IDI. The investigators will talk about all the issues related to women's participation in the study, will ask them if they understand it and if they want to participate. Their verbal consent will be recorded. |
| **Psychosocial support for interview participants** |  |  |  |  |  |  |  |
| **On-site resources to support psychosocial health of participants** | The FGDs will be conducted at clinics and locations with psychosocial services onsite. The study team will arrange to have a psychologist on site in case any of the participants desire to speak with a psychologist either during or following the discussion. | The FGDs and IDIs will be carried out at the Infovida Research Center or at the Local Hospital del Norte. Both centers have drop-in mental health services on site. The study will ask a psychologist to be onsite during the FGD or IDI and on-call following the FGDs and can follow up with the FGD participants or interviewee at the participants’ request. The cohort study's social worker will facilitate mental health care required by women who have participated in the FGDs on an as needed basis. | The FGDs and IDIs will be carried out at the Hospital Universidad del Norte. The study will schedule a psychologist from the drop-in mental health clinic to be onsite during the FGDs and IDIs and on call following the FGDs and IDIs and who will follow up with the participants if so requested by the participants. | The FGDs and IDIs will be carried out at the Hospital Carmen Emilia ISS. The study will schedule a psychologist from the drop-in mental health clinic to be onsite during the FGDs and IDIs and on call following the FGDs and IDIs and who will follow up with the participants if so requested by the participants. | No FGDs in person will be held. Interview participants will have the support of a social worker who works with UMA Association and is familiar with working with women affected by Zika and available via WhatsApp: Phone (whatsapp) 81-985421620. The team will make every effort to schedule her to be available during the interview, and following, and her number will be provided to the participants. | No FGDs will be carried out. Women will be provided with the contact information of a FREE online service provided by the STate of Rio de Janerio. | No FGDs in person will be held. Individual in-depth interviews will be conducted over the phone (WhatsApp) or other platform of the participant’s choice. The institution has a psychologist on staff that already supports families affected by Zika. This person will be available in case participants this help during the interviews. IPESQ will provide psychosocial support for participants after the InterviewThe staff psychologist will follow up with these women in case they need support following the interview or FGD. This individual is also a member of the study team, and has provided valuable contributions in the design of the study. |
| **Resources to support psychosocial health of participants in online interviews** | A mental health professional with master’s or higher level of training will be available via WhatsApp or phone during the online FGD or individual IDI. All participants will receive the contact information to schedule a one time, 1-2 hour mental health support call with the mental health professional, regardless of whether they finish the online FGD or IDI. Participants will schedule the call at their own convenience and the study will cover the cost of the online therapy visit. The therapy visit will not be recorded and the study will not know which participants received therapy sessions. | For potential participants living in rural areas with limited reception, phone interviews are not possible. The study site will not be able to recruit participants until after the COVID-19 pandemic is over. Mental health support provided to FGD and IDI participants will be as described above. A mental health professional will be available to support participants utilizing a telemedicine platform. Women who cannot access this will not be able to participate. | For potential participants living in rural areas with limited phone reception, phone interviews will not be possible and the study site will not be able to recruit participants until after the COVID-19 pandemic is over. Mental health support provided to FGD and IDI participants will be as described above. A mental health professional will be available to support participants utilizing a telemedicine platform. Women who cannot access this will not be able to participate. | For potential participants living in rural areas with limited phone reception, phone interviews will not be possible and the study site will not be able to recruit participants until after the COVID-19 pandemic is over. Mental health support provided to FGD and IDI participants will be as described above. A mental health professional will be available to support participants utilizing a telemedicine platform. Women who cannot access this will not be able to participate. | The participant will be informed that in case of any discomfort during or after the interview, she may be referred to psychologist free of charge. We will also make available the contact information for The social worker who works with UMA and the women are familiar with . | The participant will be informed that in case of any discomfort during or after the interview, she may be referred to psychologist free of charge. We will also make available the contact information for a free-of- charge online service provided by the state government of Rio de Janeiro. | Participants will be informed that in case they experience discomfort during or after the interview or focus group, they can contact the IPESQ psychologist, who already cares for these mothers and will be available assist them. |
| **On site resources to support psychosocial health of participants subsequent to the on site interview** | Women will be instructed to contact someone on the list of psychosocial support services that was provided to them by the study team on the day of the discussion if they experience emotional distress within the weeks following the study. | The FGD or interview will be conducted at the Infovida Research Center or at the Local Hospital del Norte. Both centers have drop-in mental health services on site. The study will ask a psychologist to be on call during and following the FGDs or IDIs and can follow up with the FGD or IDI participants if so requested by the participants. The cohort study's social worker will facilitate mental health care required by women who have participated in the FGDs or IDIs on an as needed basis. They can also contact someone from the list of psychosocial support services provided to them on the day of the focus group discussion. | The FGD will be conducted at Hospital Universidad del Norte. The study will schedule a mental health professional from the mental health clinic to provide support during and following the interview or discussion, and to be available on call if participants so request. | The FGD will be conducted at The Hospital Carmen Emilia ISS. The study will schedule a mental health professional from the mental health clinic to provide support during and following the interview or discussion, and to be available on call if participants so request. | FGD and IDI participants will be referred to psychosocial support services post FGD/IDI at the Hospital of University of Pernambuco (Hospital das Clínicas, UFPE). They will also receive information about whom to contact before the start of the focus group discussion. | No interviews or discussion groups will be held in person; all will be held online, and support will be provided as above. FGD and interview participants will be referred to psychosocial support services at Fiocruz (Rio de Janeiro) and a free-of- charge online service provide by state government of Rio de Janeiro. | IPESQ The institution has a psychologist on staff that has provided support to the families affected by Zika and leads women's support session groups and one on one mental health sessions. This person will be available in case participants need this service during the focus groups. IPESQ will also be the place of reference for women after the FGD and the psychologist will follow up with these women in case they need support post FGD. They will also receive information about whom to contact before the start of the focus group discussion. |
| **Referrals for psychosocial support** | FGD participants will be referred for psychosocial support through the hospitals, clinics and antenatal groups where they were recruited.  Contact information for psychosocial support services will be provided to all participants in a handout along with the informed consent form such that focus group discussion participants can connect with the services even if they decide to leave the focus group before the discussion finishes. This referral sheet will include instructions on how to contact the mental health care worker as well as how to contact the study team for assistance with scheduling and payment. | FGD participants will be referred to psychosocial support services at the Infovida Research Center or at the Local Hospital del Norte.  Contact information for psychosocial support services will be provided to all participants in a handout along with the informed consent form such that focus group discussion participants can connect with the services even if they decide to leave the focus group before the discussion finishes. This referral sheet will include instructions on how to contact the mental health care worker as well as how to contact the study team for assistance with scheduling and payment. | Contact information for psychosocial support services will be provided to all participants in a handout along with the informed consent form such that focus group discussion participants can connect with the services even if they decide to leave the focus group before the discussion finishes. This referral sheet will include instructions on how to contact the mental healthcare worker as well as how to contact the study team for assistance with scheduling and payment. | Contact information for psychosocial support services will be provided to all participants in a handout along with the informed consent form such that focus group discussion participants can connect with the services even if they decide to leave the focus group before the discussion finishes. This referral sheet will include instructions on how to contact the mental healthcare worker as well as how to contact the study team for assistance with scheduling and payment. | FGD participants will be referred to psychosocial support services at the Hospital of University of Pernambuco (Hospital das Clínicas UFPE). | FGD and IDI participants will be referred to psychosocial support services through Fiocruz (Rio de Janeiro) and a free-of- charge online service provide by state government of Rio de Janeiro.  . | FGD participants will be referred to psychosocial support services at Instituto Paraibano de Pesquisa Professor de Joaquim Amorim Neto (IPESQ). |
| **Additional ethical concerns** |  |  |  |  |  |  |  |
| **Special considerations for sensitive research with vulnerable populations** | Dr. Edna Acosta Pérez is an Assistant Professor at the Division of Community Services of the Center for Evaluation and Sociomedical Research, Graduate School of Public Health (dsc-cies.org) and a Researcher at the Behavioral Sciences Research Institute (bsri.rcm.upr.edu), and the Puerto Rico Clinical and Translational Research Consortium (prctrc.rcm.upr.edu) all at the Medical Science Campus, University of Puerto Rico. Dr. Acosta has over 14 years’ experience conducting research with vulnerable and hard to reach populations including conducting research in the areas of HIV, gender issues, violence, sexuality and mental health. Dr. Acosta is a certified Community Based Participatory Researcher and has conducted focus group discussions and qualitative research among these vulnerable populations. | The FGDs will be carried out in a private room at the Infovida Research Center or at the Local Hospital del Norte, depending on what is most convenient for the majority of FGD or IDI participants. In each of these sites, the informed consent will be administered in a private office. there will be an office for the informed consent process in a private area. In each of these sites there is a room with a capacity of 10 people where the FGD will be conducted. The AEDES Network research group, the doctors, and Social worker have had experience in conducting studies with vulnerable populations. She is driving the cohorts of pregnant women and children of Zikalliance and also the cohort assembled during the Zika outbreak. In particular, Physiotherapy, PhD in Public Health, has extensive experience in qualitative research. Members of the Emory University team, with expertise in qualitative research, will conduct training in moderating FGDs and in-depth interviewing. | The FGDs will be carried out in a private room at the Hospital Universidad del Norte depending on what is most convenient for the majority of FGD, and the IDI, participants. In each of these sites, the informed consent will be administered in a private office. There will be an office for the informed consent process in a private area. In each of these sites there is a room with a capacity of 10 people where the FGD will be conducted. Currently, no study personnel has experience in conducting FGD, will have to discuss this issue in INS. Members of the Emory University team, with expertise in qualitative research, will conduct training in moderating FGDs and in-depth interviewing. | The FGDs will be carried out in a private room at the Hospital Carmen Emilia ISS depending on what is most convenient for the majority of FGD, and the IDI, participants. In each of these sites, the informed consent will be administered in a private office. There will be an office for the informed consent process in a private area. In each of these sites there is a room with a capacity of 10 people where the FGD will be conducted. Currently, no study personnel has experience in conducting FGD, will have to discuss this issue in INS. Members of the Emory University team, with expertise in qualitative research, will conduct training in moderating FGDs and in-depth interviewing. | In Recife the project will be carried out by a researcher from Oswaldo Cruz Foundation - Aggeu Magalhães Institute (Fiocruz IAM) with experience in conducting qualitative research with vulnerable populations in contexts of violence and poverty. Also, the researcher has experience conducting FGDs among these populations. Fiocruz IAM is a research/teaching facility that has rooms equipped with computers and recording devices for conducting the FGDs and IDIs. | In Rio de Janeiro, the project will be undertaken by researchers from Oswaldo Cruz Foundation (Fiocruz) with experience in conducting qualitative research with vulnerable populations in contexts of violence and poverty. The National School of Public Health Sergio Arouca at Fiocruz houses the Centro de Saúde Escola Germano Sinval Faria, part of the Brazilian Public Health system. It is a primary health care centre that is located within the Fiocruz Campus, in the same building as the study team. This building is equipped with rooms where FGDs or IDIs could be conducted with privacy for participants. | In Campina Grande the project will be undertaken at the Instituto Paraibano de Pesquisa Professor de Joaquim Amorim Neto (IPESQ) by Dr. Adriana Melo. Dr. Melo is a neonatologist and maternal health clinician and researcher and the of President of IPESQ. She has experience in conducting research on infectious diseases and pregnancy, with an emphasis on Zika virus and congenital Zika infection and arboviruses (including microcephaly). In 2016 she published the first description of congenital Zika ultrasound findings in the world. IPESQ is equipped with a room where the FGD can be developed with privacy for participants. IPESQ has on-site private meeting rooms where FGDs and IDIs may be conducted privately. Members of the study team will conduct the focus group discussions and in-depth interviews, with the support and guidance of members of the Emory University team who have expertise in qualitative research, and will conduct training in moderating FGDs and in-depth interviewing. |
| **Incentives for in-person interview participation** | FGD participants will be offered a $40 stipend for babysitting services if needed and $30 to cover transportation to and from the FGD. Participants will receive refreshments during the FGD. No financial incentive will be provided. | FGD participants will be offered a $20 stipend for babysitting services and $30 for transportation to and from the FGD. Participants will receive refreshments during the FGD. No financial incentive will be provided. | FGD participants will be offered a $20 stipend for babysitting services and $30 for transportation to and from the FGD. Participants will receive refreshments during the FGD. No financial incentive will be provided. | FGD participants will be offered a $20 stipend for babysitting services and $30 for transportation to and from the FGD. Participants will receive refreshments during the FGD. No financial incentive will be provided. |  |  |  |
| **Incentives for online interview participation** | Participants will receive a $10 phone credit to support their participation in the online IDI or FGD. Babysitting support cannot be provided because of concerns related to the ongoing COVID-19 pandemic | No support will be provided for participation in online FGDs or telephone interviews. | No support will be provided for participation in online FGDs or telephone interviews. | No support will be provided for participation in online FGDs or telephone interviews. | Research participants will not be credited for the time that they are asked to use their phones for participation in the study. Babysitting support cannot be provided because of concerns related to the ongoing COVID-19 pandemic | Research participants will not be credited for the time that they are asked to use their phones for participation in the study. Babysitting support cannot be provided because of concerns related to the ongoing COVID-19 pandemic | Research participants will not be credited for the time that they are asked to use their phones for participation in the study. Babysitting support cannot be provided because of concerns related to the ongoing COVID-19 pandemic |
| **Recruitment dates** | April 9 2020 to November 1 2021 | February 9^th^, 2021, to June 24^th^ 2021 | February 9^th^, 2021, to June 24^th^ 2021 | February 9^th^, 2021, to June 24^th^ 2021 | November 13^th^ 2020 to July 7^th^ 2021 | November 13^th^ 2020 to July 7^th^ 2021 | November 13^th^ 2020 to July 7^th^ 2021 |
