# Supplementary material for: “I found out about Zika virus after she was born.” Women’s experiences of risk communication during the Zika virus epidemic in Brazil, Colombia, and Puerto Rico
Source: PLOS Glob Public Health. 2024 Jun 12;4(6):e0002808. doi: 10.1371/journal.pgph.0002808 (PMC11168637; doi:10.1371/journal.pgph.0002808)
Supplement: S2 Table — (DOCX) [file pgph.0002808.s002.docx]

**S2 Table. Supporting quotes in original Spanish or Portuguese and English translation**

| **Theme** | **Quote number** | **Original language** | **English-language translation** | **Participant** |
| --- | --- | --- | --- | --- |
| Diagnostic testing for Zika infection and communication of results | 1 | A mí a los 2 meses, a mí me dio Zika. Yo me broté y yo fui a urgencias, para que me hicieran la prueba, pero incluso yo fui a urgencias y me dijeron tome acetaminofén y ya, para la casa, nada más, o sea me dijeron que no. Yo solicité el examen y me dijeron que no, entonces yo le dije que me daba miedo porque estaba embarazada y quería hacerme el examen y me dieron que no, que no había problema, que tomara acetaminofén y ya nada más, pero pues sí, a uno le da como la incertidumbre, pero pues ya estaba embarazada y todo y pues la verdad en el momento que a mí me dio, yo lo tome como pasajero, se me pasó y ya no volví a pensar en eso, eso fue ya casi a los 7 meses fue que empezó ya digamos como que a sospechar algo y todo eso. Antes fue un embarazo totalmente normal. | At 2 months, I got Zika. I had an outbreak and I went to the emergency room to get tested, but even I went to the emergency room and they told me to take acetaminophen and that's it, just go home, that is, they told me no. I requested the test and they told me no, so I told him that I was scared because I was pregnant and wanted to do the test and they told me no, that there was no problem, that I should take acetaminophen and nothing else, but yes, to one gives you the uncertainty, but well I was already pregnant and everything and well the truth at the time it hit me, I took it as a passenger, it passed me by and I didn't think about it again, that was almost 7 months was when he began, let's say, to suspect something and all that. Before, it was a completely normal pregnancy. | Mother of a child with CZS, Colombia, participant NEIVA004 |
|  | 2 | Quando eu engravidei de [nome da criança], eu tava com 12 semanas e eu comecei ... eu desenvolvi uma coceira muito grande e essa coceira ficou tão ... me incomodou tanto que eu precisei procurar a emergência e lá na emergência ... e eu perguntei o que isso poderia afetar meu bebê, a doutora disse que nada, que era feito uma dengue e que iria passar. Me passou um anti alérgico, algumas ultrassons. Tomei o anti alérgico, passei ainda quatro dias com essa coceira, fiz as ultrassons e tava tudo normal. | When I got pregnant with [child's name], I was 12 weeks and I started… I developed a really bad itch and this itch got so… bothered me so much that I had to go to the ER and then to the ER. .. and I asked him what could this affect my baby, the doctor didn't say anything, that it was like dengue and that it was going away. He gave me an allergy medicine, some ultrasounds. I took the allergy medicine, I had this itch for four days, I had the ultrasounds and everything was normal. | Mother of a child with CZS. Brazil, participant BRID_SPECP12 |
|  | 3 | Las primeras pruebas.. pues todo salió bien. Un día mi cita de rutina del ginecólogo y el me…yo le digo, le hago el acercamiento de que tenía un rash (sarpullido) en todo el cuerpo. Entonces el doctor lo que me recetó un jabón para la piel como para calmarme el picor y lo que era el rash (sarpullido) y me dio unos antibióticos. Entonces me dijo: “Te vamos a repetir la prueba del Zika porque ya vas a estar entrando a lo que sería el tercer trimestre para repetirla antes de [que des a luz]”. Cuando llegan los resultados ahí me dicen: “Mamá saliste Zika positivo”. | The first tests... well, everything went well. One day I had a routine appointment with the gynecologist and he… I told him, I told him that he had a rash all over his body. So the doctor prescribed a soap for my skin to calm the itching and what the rash was and gave me some antibiotics. Then he told me: “We are going to repeat the Zika test because you are going to be entering what would be the third trimester to repeat it before [you give birth].” When the results come in, they tell me: “Mom, you came out positive for Zika.” | Mother of a child with CZS. Puerto Rico, participant ID304 |
|  | 4 | Me decía, “aquí en todos estos saliste negativo, en el único que saliste positivo fue Zika, así que tú tienes Zika”. De allí, yo abrí los ojos bien grandes, a pesar de que ya me lo sospechaba, pues tenía un poquito de fe que no, que fuera solamente una bobería lo que hubiese tenido y no fuera eso. Entonces allí ella me dijo el protocolo [de hospital público]. Que entonces ahora yo tenía que ir a un perinatólogo que se está especializando en mamás embarazadas con Zika. | He told me, “here in all these you came out negative, in the only one you came out positive was Zika, so you have Zika”. From there, I opened my eyes very wide, despite the fact that I already suspected it, because I had a little faith that it wasn't, that it was just silly what I had had and it wasn't that. So there she told me the [public hospital] protocol. So now I had to go to a perinatologist who is specializing in pregnant moms with Zika. | Mother of a child with CZS. Puerto Rico, participant ID305 |
| Explanation of ZIKV test results and associated uncertainty | 5 | Yo creo que a uno también le tienen que explicar, de seguro uno no sabe que efectos trae, porque a mí, por ejemplo, solo me dijeron que tenía zika y que era una enfermedad que podía afectar al bebe, que lo deforma, eso fue todo. | I think that they also have to explain to one, surely one does not know what effects it brings, because to me, for example, they only told me that I had Zika and that it was a disease that could affect the baby, that it deformed it, that was all. | (Mother of a child without CZS. Colombia, participant NOCZS008) |
|  | 6 | Ellos (personal sanitario) dijeron que era por Zika, enseguida dijeron que lo asociaron con el Zika, pero en sí, no hay un examen de sangre que certifique que es por el Zika, o sea como nació en el mes de los niños del Zika a él lo metieron ahí con ellos, pero no hay un examen de sangre que me diga que si es por Zika. | They (health personnel) said it was due to Zika, they immediately said that they associated it with Zika, but in itself, there is no blood test that certifies that it is due to Zika, that is, since he was born in the month of Zika children, they put him there with them, but there is no blood test that tells me if it is due to Zika. | (Mother of a child with CZS. Colombia, participant BQUILLA002) |
|  | 7 | Então, o resultado eu já sabia, porque depois que ela nasceu vimos que ela tinha microcefalia. Eu já sabia que era de Zika, o resultado era o menos importante, o resultado positivo de Zika era menos importante, como eu já sabia eu sabia que tinha microcefalia… | So, I already knew the result, because after he was born we saw that he had microcephaly. I already knew it was from Zika, the result was the least important, the positive result for Zika was less important, as I already knew, I knew that I had microcephaly. | (Mother of a child with CZS. Brazil, participant SPECP01) |
|  | 8 | Esse resultado quando veio eu já sabia né, que ele já estava com 1 ano e pouquinho, já estava com 1 ano e 4 meses mais ou menos [nome da criança] a gente já estava até fazendo tratamento. ... fez a tomografia e veio pra médica, ai foi ela disse que [nome da criança] tinha paralisia cerebral e microcefalia e tinha as calcificações né, ai pronto, daí eu já sabia que [nome da criança] tinha microcefalia, ai depois com 1 ano eu fui descobrir que realmente era, foi pelo Zika. | When this result came out I already knew, he was already a year and a bit old, he was 1 year and 4 months old more or less [child's name], we were already in treatment. … she had the tomography done and came to the doctor, so she (doctor) said that [child's name] had cerebral palsy and microcephaly and had calcifications, well, then, she already knew that [child's name] had microcephaly, then after In 1 year I found out that it really was, it was because of Zika. | (Mother of a child with CZS. Brazil, participant SCGBS06) |
|  | 9 | E: - E como foi explicado o resultado do exame de Zika? Você se lembra do resultado?  C: Ah, eu lembro que foi positivo, aí o médico me explicou lá também né, conversei com o médico que me atendeu naquele dia e depois conversei com o meu médico. Aí ela disse “olha mãe, não fique nervosa com as coisas que você está vendo na televisão, você está com Zika, mas se Deus quiser, vai ficar tudo bem com seu bebê... ultra-som, então não se preocupe porque você já está no final da gravidez, então com certeza nada vai acontecer com seu bebê. Aí eu fiquei nervosa, porque esses dias meu marido estava trabalhando e eu tive que ir sozinha no hospital, aí eu saí de lá chorando né, porque você fica nervosa, mesmo que a pessoa fale que vai dar tudo certo, você fica apreensiva Hã. Aí eu fiquei muito apreensiva, mas confiante de que tudo ia dar certo, entendeu? | E: And how was the result of the Zika test explained? Do you remember the result?  P: Ah, I remember it was positive, so the doctor explained to me there too, right, I talked to the doctor who treated me that day and then I talked to my doctor. Then he told me “look mom, don't get nervous about the things you're seeing on television, you're with Zika, but God willing, everything will be fine with your baby... in one more week you're going to have an ultrasound, so do not worry because you are already at the end of the pregnancy, so surely nothing will happen to your baby. So I got nervous, because these days my husband was working and I had to go to the hospital alone, so I left crying, right, because you get nervous, even if the person says that everything is going to be fine, you get apprehensive. Hey. So I was very apprehensive, but sure that everything was going to be okay, you know? | (Mother of a child without CZS. Brazil, participant BRID_NRJEP15) |
|  | 10 | P: Un médico, supuestamente del [nombre de hospital] me llamó a informarme que era positiva y que me tenía que seguir cuidando o si sentía alguna complicación o alteración o más fiebre o algo así que no se pudiera bajar con las pastas que tenía que ir directamente al hospital  E: Entonces le han dicho, bueno su resultado es positivo y ¿el médico le explicó qué significaba ese resultado positivo?  P: No, sólo me dijeron que positivo y que tenía que cuidarme, pues a mí no me afecta mucho porque yo ya el embarazo estaba muy avanzado, ya mi hijo se había desarrollado, ya estaba bien madurándose  E: ¿le dijeron algo de la posibilidad de que el virus afectará a su bebé?  P: Sí, pero como le digo, ya no era mucho porque supuestamente el niño ya estaba completamente formado  E: ¿Eso se lo explicó el médico cuando le dijo lo del resultado?  P: Sí, pero igual no descartaban complicaciones en el feto pues porque iba a tener un porcentaje  E: ¿de posibilidad de que algo estuviera mal sí?  P: pero por el momento ellos decían que no | P: A doctor, supposedly from the [name of hospital] called me to inform me that I was positive and that I had to continue taking care of myself or if I felt any complication or alteration or more fever or something like that that could not be lowered with the medication that I had to go straight to the hospital  E: So they told you, well, your result is positive, and did the doctor explain what that positive result meant?  P: No, they only told me that it was positive and that I had to take care of myself, because it does not affect me much because the pregnancy was already very advanced, my son had already developed, he was already maturing well  I: Did they tell you anything about the possibility that the virus will affect your baby?  P: Yes, but as I say, it wasn't much anymore because supposedly the child was already fully formed  E: Did the doctor explain that to you when he told you about the result?  P: Yes, but they still did not rule out complications in the fetus because it was going to have a percentage  I: Of the possibility that something was wrong, yes?  P: but at the moment they said no | (Mother of a child without CZS. Colombia, participant NOCZS005) |
|  | 11 | E: Me hablaste de que la doctora te dijo que a lo mejor el bebé no iba a tener complicaciones, ¿verdad? porque estabas en tus ocho meses de embarazo, pero, ¿qué tan segura estaba esa doctora acerca del resultado del zika para ti?  P: Más o menos.  E: Y, ¿te explicó algo del por qué o más o menos?  P: No  E: ¿Te explicó algo acerca sobre la posibilidad de que el virus afectara a tu bebé?, Si lo hizo, ¿qué te explicó?  P: Que podía venir con microcefalia, deformaciones y tal vez no llegar a los tres años. | E: You told me that the doctor told you that the baby might not have any complications, right? because you were eight months pregnant, but how sure was that doctor about the zika result for you?  Q: More or less.  E: And, did he explain to you something about why or more or less?  Q: no  E: Did he explain anything to you about the possibility of the virus affecting your baby? If he did, what did he explain to you?  P: That he could come with microcephaly, deformities and maybe not reach three years. | (Mother of a child with CZS. Puerto Rico, participant ID301) |
| Diagnosis of Zika-related neurological disorder | 12 | C: quando eu engravidei que eu fui infectada por esse vírus, me disseram só que era chikungunya, que não sabia o que poderia acontecer. Informei que estava gravida e então ninguém soube me informar o que poderia acontecer, e assim, tive pequenos sangramentos, e segui adiante com a gestação, peguei na décima segunda semana de gestação, então como ninguém sabia informar nada sobre isso, não tinha nem como saber como que seria a gravidez, feto, pós, nem nada, foi tudo muito surpresa pra mim, só descobri mesmo zika vírus, depois que ela nasceu, que foi quando começaram a nascer também no Nordeste todas elas com microcefalia, e que começaram a fazer os exames, viram que de fato era microcefalia pelo zika vírus mesmo. | When I got pregnant that I was infected with this virus, they only told me it was Chikungunya, that they didn't know what could happen. I reported that I was pregnant and then nobody could tell me what could happen, and then, I had a little bleeding, and I went ahead with the pregnancy, it came out in the twelfth week of pregnancy, so since nobody knew anything about it, there was no way to knowing what the pregnancy, fetus, postpartum, or whatever would be like, everything was very surprising for me, for me I only found out about the Zika virus after she was born, which was when they also began to be born in the Northeast, all with microcephaly , and when they began to do the tests, they saw that it was actually microcephaly due to the Zika virus. | (Mother of a child with CZS. Brazil, participant SRJEP03) |
|  | 13 | Entonces él (ginecólogo) sacaba la cuenta, sacaba la cuenta. “Es que no me da, no me da”, “la cabeza no está del tamaño que debe tener para el tiempo de embarazo.” Cuando él miraba la ecografía nada, nada, llamó a otro primatólogo que estaba ahí, “de pronto yo me equivoqué, de pronto algo hice mal, vamos a confirmar.” Y él volvió, volvió y el otro señor también miraba y nada, definitivamente no. Luego llegó otro y también dice lo mismo. Entonces ya ellos se quedaron, así como que no habían tenido, así como, como muchos casos así, así cerquita, empezaron ahí sí a preocupar, ahí sí a explicarme lo que había pasado. Este pasa esto, me preguntó, ¿tú tuviste fiebre?, ¿tú tuviste algún síntoma de eso? Él me hace las preguntas y yo sí, cuando tenía 8 semanas de embarazo me dio fiebre, manchitas así, dolor en las articulaciones, dolor de cabeza, pero nunca me dijeron nada, yo fui y nunca me dijeron nada de que había que mirar, hacer un seguimiento más profundo, o sea nada diferente a una ecografía. Entonces, el doctor enseguida me dijo que no, que la bebé lo más probable era que venía con una microcefalia porque, ¿cómo es?, la cabeza no estaba, su cerebrito no estaba desarrollado completamente, estaba más pequeño del tiempo, que para el tiempo de embarazo que yo tenía, no tenía el tamaño que era y entonces me mandó una serie de exámenes como para descartar…. Fue la primera vez que me mandaron a hacer el examen de sangre para saber si me había dado Zika o no, ese mismo día me lo hicieron. | Then he (gynecologist) calculated [the head circumference], and calculated it. "It just doesn't work, it doesn't work, the head is not the size it should be for the time of pregnancy." When he looked at the ultrasound nothing, nothing. He called another primatologist who was there, "maybe I was wrong, maybe I did something wrong, let's confirm." And he came back, came back and the other man also looked and nothing, definitely not. Then another one came and also says the same. So they stayed, just like they hadn't had, just like, like many cases like that. So close, they began to worry, then to explain to me what had happened. “This is what happened.” He asked me, “did you have a fever? Did you have any symptoms of that?” He asks me the questions and I did. When I was 8 weeks pregnant I got a fever, spots like that, pain in the joints, a headache, but they never told me anything. I went and they never told me anything that I had to look at, do a deeper follow-up, that is, nothing different from an ultrasound... So, the doctor immediately told me no, that the baby most likely came with microcephaly because, how is it? The little head was not, her brain it was not fully developed, it was smaller than the time, that for the time of pregnancy that I had, it did not have the size that it was. And so he sent me a series of tests to rule out…. It was the first time they sent me to do a blood test to find out if I had had Zika or not, that same day they did it. | (Mother of a child with CZS. Colombia, participant BQUILLA001) |
|  | 14 | P: … haciéndome la ecografía a él (ginecólogo) no le cuadraban unos, unos, eh.. unas medidas, me dijo él “no, es que de pronto mi ecógrafo se dañó”, volvió y lo hizo, volvió, borró, apagó y cuando me dice “tenemos que hacerle unos exámenes más a fondo, de pronto mi ecógrafo me está fallando y pues tengo que hacerle algo”, entonces yo le dije ¿cómo así? Entonces me decía que es que no le cuadraban unas medidas del perímetro cefálico de mi hijo, eh en su cabecita, “me explica más en español” le decía yo, y que pues teníamos que realizar unos exámenes, entonces me da la formula, me da las órdenes para yo hacérmelas por la EPS y pues eso fue en cuestión de 15 días, por la prioridad, él sí le dio prioridad y a los 15 días me la realizan.. el médico pensando que yo ya sabía el diagnóstico, me dijo “sí, efectivamente tiene el niño sus calcificaciones, tiene el niño microcefalia y tatata.. tatata” y me dice todo como de una, no? | Doing the ultrasound on him (gynecologist) some, some, uh... some measurements did not add up, he told me "no, it's that suddenly my ultrasound machine was damaged," he came back and did it, came back, erased, turned off and when he told me, “we have to do some more in-depth tests, suddenly my ultrasound machine is failing me and well I have to do something to him”, then I told him how come? Then he told me that some measurements of my son's head circumference did not add up, eh in his little head, "he explains more in Spanish" I told him, and that well we had to carry out some tests, then he gives me the formula, I He gave the orders for me to have them done by the EPS and, well, that was in a matter of 15 days, because of the priority, he did give it priority and after 15 days they did it for me...the doctor, thinking that I already knew the diagnosis, told me, “yes, indeed the child has his calcifications, the child has microcephaly and tatata .. tatata” and he tells me everything as one, right? | (Mother of a child with CZS. Colombia, participant BUCA002) |
|  | 15 | E: Quando você teve esse diagnóstico de microcefalia o que foi dito para você?  C: Quando eu tinha... eu tinha assim, a gente já estava acompanhando, com medo, mas eu tinha muita fé que não ia acontecer nada. Ainda estava na sala de ultrassom né, quando veio a diferença, ela disse, realmente agora tinha diferença de perímetro cefálico, e lá a gente já sabia o que era microcefalia, mas ela também não explicou muito. Foi o primeiro caso dela... aqui no Rio, foi o primeiro caso desse surto que ela estava cuidando de uma mulher, uma criança, né, com microcefalia. Então ela também não me explicou muito, foi só na mídia. | We were already following it [the outbreak], with fear, but I had great faith that nothing was going to happen. I was still in the ultrasound room, right, when the difference came, she said, really now there was a difference in head circumference. And there we already knew what microcephaly was, but she didn't explain much either. It was her first case... here in Rio, it was the first case in this outbreak that she was caring for a woman, a child, no? with microcephaly. So she also didn't explain much to me, it was only on the media. | (Mother of a child with CZS. Brazil, BRID_SRJEP06.) |
|  | 16 | E: Você ouviu o que poderia acontecer se a gestante tivesse Zika?  P: Quando eu ouvi eu fiquei com medo, porque quando eu vi na televisão que as mães estavam tendo esses filhos com microcefalia, e era uma coisa que eu nunca tinha visto falar sobre microcefalia, nunca na minha vida, eu fiquei com muito medo.  Lá estava eu, foi quando ela (médica) falou "seu filho tem microcefalia", ela só foi falar isso, aí eu nem deixei ela fazer o ultrassom porque eu estava desesperada na hora, né? como se meu mundo tivesse desmoronado naquele momento. Eu não sabia o que era microcefalia, e ela nem me explicou nada, se ela tivesse me explicado, "ah, é assim", talvez eu até aceitasse ali naquela época... estou tentando me livrar da mágoa, mas no começo eu fiquei muito brava com aquela médica, queria colocar toda a culpa nela, sabe, como se fosse ela que tivesse destruído o meu sonho. | E: Did you hear or what could happen if a pregnant woman had Zika?  P: When I used to talk about microcephaly, because when I saw on television that I was most likely to have these children with microcephaly, it was something that I had never seen talk about microcephaly, never in my life, I was very afraid.  She was there, it was when she (doctor) said "she was a child with microcephaly", she was just doing it, so she didn't need to do anything because she was desperate at the time, right? I didn't know what microcephaly was, and she didn't explain anything to me, she explained it to me, "oh, it's like that", maybe I used oil at that time... I'm tempted to get rid of the magic, but I don't eat and eat Very brave with that doctor, I wanted to place all the blame on her, you know, how she felt that she was destroyed or my dream. | (Mother of a child with CZS. Brazil, participant BRID_SCGBS01) |
|  | 17 | E: ¿Con quién estaba ese día que le explicaron todo sobre su bebé?  P: Con mi esposo… Resulta que el primer día fui con mi niña mayor a conocer a la bebé, pero como no esperaba que me dieran esa noticia, me enfermé, ¿no? Entonces me puse muy mal y me fue cuando el médico me explicó, porque comencé a verle la cara, vi que estaba hablando con el otro médico y que vi que algo no les cuadraba, comencé a sospechar. Cuando me explicaron la situación, no estaba bien, me enfermé y salí de la consulta. Me bajé y me fui a la calle, porque me puse muy mal, entonces esto... y no quería que mi niña mayor viera lo que estaba pasando, no quería que se diera cuenta, porque yo no sabía cómo explicárselo. Me sentí mal porque ella había ido a conocer a la bebé y no se la podíamos mostrar. | E: Who were you with that day when they explained everything about your baby?  P: With my husband… It turns out that the first day I went with my oldest girl to meet the baby, but since I didn't expect to be given that news, I got sick, right? Then I got very sick and it left when the doctor explained it to me, because I began to see her face, I saw that she was talking to the other doctor and that I saw that something was wrong with them, I began to suspect. When they explained the situation to me, I was not well, I got sick and left the consultation. I got out and went to the street, because I got really sick, so this... and I didn't want my older girl to see what was happening, I didn't want her to notice her, because I didn't know how to explain it to her. I felt bad because she had gone to meet the baby and we couldn't show it to her. | (Mother of a child with CZS. Colombia, participant BQUILLA001) |
|  | 18 | … ellos [médicos] me decían siempre que eso era una enfermedad nueva, que era una enfermedad que no se sabía, que ellos no tenían una certeza de que iba a pasar, que en todos los niños se estaba comportando de maneras diferentes, que tenía que estar preparada para lo bueno y para lo malo, que no me hiciera tantas ilusiones. | … they [doctors] always told me that this was a new disease, that it was a disease that was not known, that they were not certain that it was going to happen, that in all the children it was behaving differently, that it had to be prepared for the good and for the bad, that I not have so many illusions. | (Mother of a child with CZS. Colombia, participant BUCA002) |
|  | 19 | Había el boom, el brote fue como allá en Brasil y los medios informaban que el problema que estaban teniendo las madres cuando… era el problema de los bebés mayormente que estaban teniendo problemas cuando nacían. Entonces ella [médica] lo que, me dijo fue que me mandó todo el tiempo al perinatólogo. No me hizo un sonograma regular, porque el perinatólogo lo ve todo más específico, si hay algún defecto o una ineficiencia en el bebe. Por eso fue que el cuidado fue más intenso comparado con mi otro embarazo que fue normal. Fue bien intenso y ella fue bien meticulosa. Fue bien precisa en todo. | There was a boom, the outbreak was like there in Brazil and the media reported that the problem that the mothers were having when… was the problem of the babies, mainly that they were having problems when they were born. So what she [doctor] told me was that she sent me to the perinatologist all the time. She did not give me a regular sonogram, because the perinatologist sees everything more specifically, if there is any defect or inefficiency in the baby. That's why the care was more intense compared to my other pregnancy, which was normal. It was very intense and she was very meticulous. She was very precise in everything.  All interviewees with a child affected by Zika described the personal challenges they faced when receiving information about their child’s condition. However, those who received information in private, in a calm and considerate manner, felt that the deliberate, thoughtful way the information was provided helped them cope with the difficult situation. | (Mother of a child with CZS. Puerto Rico, participant ID305) |
| Interaction between delivery of information and women’s cultural beliefs | 20 | Me llevó a la oficina de él (doctor), fuera de donde me hicieron la ecografía, nos sentamos, nos habló, nos habló, nos dijo las cosas eh digamos positivas, las cosas negativas que podría tener la condición de la niña, nos fue sincero desde el principio, porque él no me dijo, “no, la niña viene mal y no vas a poder hacer nada”. Nos dijo la niña no viene bien, pero tampoco te puedo decir cuál es la voluntad de Dios. Cuando ya le hablan a uno así, aunque está uno con el dolor, es diferente a que le digan: “no, la niña viene mal.. no, mire tiene que irse para tal parte y vaya y hágase esto, no sé, ya es decisión de ustedes”. Pero él, sin embargo, nos dijo: ustedes tienen derecho a una interrupción del embarazo si quieren, si quieren, no es obligación.... como le digo, [el doctor] fue muy sutil para decir las cosas, entonces yo digo que para explicarle eso, para que uno no quede tan tan, como tan traumado. | He took me to his (doctor's) office, outside where they did the ultrasound, we sat down, he talked to us, he talked to us, he told us the things, let's say positive, the negative things that the girl's condition could have, it was sincere from the beginning, because he didn't tell me, “no, the girl is bad and you won't be able to do anything”. He told us the girl is not doing well, but I can't tell you what God's will is either. When they talk to you like that, even though you're in pain, it's different from being told: "no, the girl is bad... no, look, you have to go somewhere and go and do this, I don't know, now it is your decision”. But he, however, told us: you have the right to an interruption of the pregnancy if you want, if you want, it is not an obligation.... as I say, [the doctor] was very subtle in saying things, so I say that to explain that to him, so that one does not remain so, so traumatized. | (Mother of a child with CZS. Colombia, participant BQUILLA001) |
|  |  |  |  |  |
|  |  |  |  |  |
|  |  |  |  |  |
