# Supplementary material for: “I found out about Zika virus after she was born.” Women’s experiences of risk communication during the Zika virus epidemic in Brazil, Colombia, and Puerto Rico
Source: PLOS Glob Public Health. 2024 Jun 12;4(6):e0002808. doi: 10.1371/journal.pgph.0002808 (PMC11168637; doi:10.1371/journal.pgph.0002808)
Supplement: S3 Table — (DOCX) [file pgph.0002808.s003.docx]

Appendix Table S3. Codebook

| **Code** | **Description** |
| --- | --- |
| **Actual Communication of Results** | Use for any discussion about how women learned about their ZIKV test results not mentioned in the child codes. This includes positive and/or negative tests or ultrasound. |
| **Actual Communication of Test Results** | Use for any description of how women were told about their ZIKA test result (or the lack of them). Use for description about the experience. E.g. who told them, where they were, how they were told, who was with them.   Also use for description of any discussion with the provider about the possibility (chance) that ZIKA could affect the pregnancy and/or fetus/baby. This includes the chance that ZIKA might affect the preganancy, fetus or infant/child. Also use for mention that this disucssion DID NOT take place or this information was not communicated to the participant. |
| **Actual Negative test / ultrasound result / Resultados reales test negativo /** | Use for discription about the doctor's certainty that the test result was negative. Include description of WHY the doctor said they thought it was negative, include discussion of their certainty/incertainty of the diagnosis. Include mention of possiblity of negative being positive.   Double code with Uncertainty. |
| **Actual Positive test / ultrasound result / Resultados reales test positivo /** | Use for discription about the doctor's certainty that the test result was positive. Include description of WHY the doctor thought it was positive, and description of what a positive test would mean. Include possible impact to the pregnancy, fetus, infant/child. |
| **Provider Certainty/Uncertainty** | Participant's description of healthcare provider's certainty or uncertainty about the accuracy of a positive or negative test result. |
| **Challenges** | Use for any discussion about the CHALLENGES of having a baby or child affected by ZIKV/with CZS. |
| **CZS Description** | Use for description of HOW Zika affected their infant/child. |
| **Emotions, Feelings, Uncertainty** | Use for indication of how women feel about their situation, their fetus/infant/child's situation. To any many mention of their feelings in the past or present. This includes mention of being uncertain about possible outcomes or information. |
| **Ethics** | Use for mention of things related to ethics, including use of data and consent, ways women were informed about their test results, what women were told were told about their test results, voluntary participation in studies, if they were told that their data would be used, etc. |
| **Faith** | Use for mention of spiritual faith. |
| **Good Quotes** | Use for quotes that can be used for examples in the manuscripts or for providing guidance to providers. |
| **Memory / Passage of Time** | Use for mention that a lot of time has passed since Zika, they don't remember or it is hard/difficult to remember. |
| **Recommendations** | Use for mention of recommendations made by the provider during or after the pregnancy. |
| **SES** | Use for discussion about aspects of their SES that might impact their understanding about or abiltiy to prevent Zika. Also use for how SES impacts their ability to care for a CZS-affected child. |
| **Stigma** | Use for mention of Zika-related stigma, or fear of stigma related to ZIKV or having a CZS-affected child. |
| **Ideal Communication of Results** | Use for any discussion about the ideal way to communicate results. |
| **First heard about Zika** | Use for discussion of when participant first heard or learned about Zika. This includes WHEN they hear about it.  When was the first time you heard about Zika? |
| **Zika Experience** | Use for any discussion about Zika (e.g. the symptoms they had, how they felt, what they did) that is not included in any of the codes. |
| Additional comments | Use for final additional comments that participants share.   Double code with any other relevant codes. |
| Comments for Researchers | Use for one thing participants would like to tell researchers about ZIKA.   Double code with any other relevant codes. |
| Zika Knowledge | Use this code for any discussion of participant knowledge about Zika that is not related to one of the codes below. |
| Zika Perceptions of Risk | Use for any discussion about risk or perception of risk of Zika that is not included in the sub/child codes below. |
| Risk to Pregnancy/Fetus (General) | Use for discussion of what they heard about Zika infection in pregant women from any source and what could happen to the pregnancy and/or fetus. Also use for comparison to other diseases. |
| Defining ZIKV risk through comparison with other illnesses | Any social/cultural/biomedical description of illness during pregnancy that could have negative consequences on reproductive health, i.e. STORCH infections (Syphilis, Toxoplasma,Rubella, CMV, HIV etc.); arboviral diseases (DENV, CHIKV...), and others (genetic disorders etc.). |
| Risk to Infant/Child | Use for discussion of what they heard about Zika infection in pregant women from any source and what could happen to the development of their child. |
| Risk to Own pregnancy | Use for discussion of how women though their OWN pregnancy could be affected by ZIKA based on what they had heard about what could happen if a pregnant woman became infected with Zika and what that could do to their pregnancy/fetus/child's development later on. |
| Therapeutic Itinerary | Use for discussion about the pathways (journey) taken within the healthcare system to secure information, obtain care, resources, and treatment.   The term “therapeutic itinerary” (TI) refers to the search for treatment and seeks to describe and analyze individual and socio-cultural practices, in terms of the paths taken by individuals to solve their health problems, , including the logic that drives this pursuit, which is woven in multiple formal and informal networks of support to which a person belongs |
