# Supplementary material for: “I found out about Zika virus after she was born.” Women’s experiences of risk communication during the Zika virus epidemic in Brazil, Colombia, and Puerto Rico
Source: PLOS Glob Public Health. 2024 Jun 12;4(6):e0002808. doi: 10.1371/journal.pgph.0002808 (PMC11168637; doi:10.1371/journal.pgph.0002808)
Supplement: Attachment — Submitted filename: Example ICF for reviewers.docx [file pgph.0002808.s005.docx]

**Termo de Consentimento Livre e Esclarecido (TCLE)**

Prezada Participante,

Você está sendo convidada a participar da pesquisa “ **Comunicação de risco em tempos de Zika Vírus: uma investigação sobre percepções e preferências de mulheres para serem informadas sobre seu estado de infecção por Zika Vírus e a probabilidade de apresentarem resultados adversos na gravidez** ” coordenada no Brasil por Gustavo Correa Matta e Ester Paiva Souto, pesquisadores da Escola Nacional de Saúde Pública Sérgio Arouca, da Fundação Oswaldo Cruz (ENSP/FIOCRUZ).

O objetivo principal deste estudo é entender o que as mulheres que estiveram grávidas na época da epidemia de Zika pensam sobre as informações que recebem com relação ao vírus e sobre suas possíveis consequências na gravidez. Também queremos entender as suas opiniões sobre a utilização de informações anônimas de participantes de pesquisas (disponíveis, por exemplo, em prontuários, registros hospitalares, ultrassonografias) nesses estudos. Algumas vezes em pesquisas são utilizadas informações de várias pessoas que participaram de vários estudos sem incluir seus nomes ou dados que possam identificá-los. A combinação dessas informações e a análise deste material é chamada "meta-análise dos dados do participante". Gostaríamos de saber o que vocês pensam sobre isso.

O convite para a pesquisa se deve ao fato de você ter estado grávida na época da epidemia de Zika no Brasil e estar participando de um estudo deste tipo. A sua participação é voluntária, você pode decidir se quer ou não participar, bem como desistir de participar a qualquer momento da pesquisa, sem que isso resulte em qualquer problema para você. Você poderá ficar em silêncio em qualquer momento do debate em que não se sentir à vontade para falar.

Convidaremos você a participar de uma entrevista individual por telefone. A entrevista terá duração média de 60 minutos, mas podemos realizá-la em mais de uma ligação, de acordo com sua disponibilidade. Você será convidada a falar sobre sua vivência e o que foi informada sobre os riscos de ter Zika durante a gravidez. Se você concordar, o áudio desse encontro será gravado, transcrito e guardado em arquivos digitais, aos quais somente terão acesso os pesquisadores.

A sua participação na pesquisa não trará nenhum custo para você, sendo ressarcidas quaisquer despesas decorrentes da sua participação no estudo, tais como: gastos com transporte, alimentação e cuidadores para crianças. Vamos perguntar ao final da entrevista se você estaria disposta a falar conosco uma segunda vez para apresentarmos os resultados ao final do estudo. Você poderá escolher a meio de contato (WhatsApp, e-mail, etc.).

Página 1 de 3

Rubrica pesquisador: ______________

Rubrica participante: ______________

Serão tomadas as seguintes medidas para assegurar a confidencialidade e a privacidade das informações: os pesquisadores do projeto não farão uso destas informações para outras finalidades que não as previstas pelo projeto; qualquer dado que possa identificá-la será excluído na divulgação dos resultados da pesquisa; o material coletado será armazenado em arquivos digitais protegidos por pelo menos 5 anos, conforme Resolução 466/12 e orientações do CEP/ENSP, cujo acesso será restrito apenas aos pesquisadores envolvidos na pesquisa.

A qualquer momento, durante a pesquisa, ou posteriormente, você poderá solicitar do pesquisador informações sobre sua participação e/ou sobre a pesquisa, o que poderá ser feito através dos meios de contato explicitados neste Termo. Se houver algum dano você terá direito a solicitar a qualquer momento indenização, através das vias judiciais, como dispõem o Código Civil, o Código de Processo Civil e a Resolução nº 466/2012, do Conselho Nacional de Saúde (CNS) e Resolução Complementar 510/2016 do mesmo Conselho.

Os riscos em participar desta pesquisa são mínimos, decorrentes do desconforto em falar de assuntos sensíveis. Para minimizá-los, lembramos que é possível que você interrompa sua participação a qualquer tempo, ou retire seu consentimento, sem qualquer problema para você. Caso você sinta algum desconforto durante a conversa ou posteriormente, nos avise que nós iremos providenciar seu encaminhamento a um profissional específico (psicológico ou assistente social) sem custos para você ou aos serviços de saúde. Como benefícios indiretos de sua participação, você estará contribuindo para a melhora da resposta do sistema de saúde frente a epidemias como a da Zika.

**Por favor, salve uma cópia deste termo assinado, de maneira que você possa consultá-lo a qualquer momento durante a pesquisa.**

Quaisquer dúvidas relativas à pesquisa, tanto antes quanto após o início e fim do estudo poderão ser consultadas através dos telefones/e-mails e endereço colocados abaixo.

**Contato com os pesquisadores responsáveis:**

Gustavo Correa Matta (ENSP/FIOCRUZ) / (e-mail: gcmatta@ensp.fiocruz.br)

Ester Paiva Souto (ENSP/FIOCRUZ) / (e-mail: esterpaiva@gmail.com)

Em caso de dúvida quanto à condução ética do estudo, entre em contato com o Comitê de Ética em Pesquisa da ENSP. O Comitê de Ética é a instância que tem por objetivo defender os interesses dos participantes da pesquisa em sua integridade e dignidade e para contribuir no desenvolvimento da pesquisa dentro de padrões éticos. Dessa forma o comitê tem o papel de avaliar e monitorar o andamento do projeto de modo que a pesquisa respeite os princípios éticos de proteção aos direitos humanos, da dignidade, da autonomia, da não maleficência, da confidencialidade e da privacidade. O Comitê pode ser contatado no telefone (21) 2598-2863, no e-mail cep@ensp.fiocruz.br ou ainda no endereço Rua Leopoldo Bulhões, 1480 – Térreo -

Página 2 de 3

Rubrica pesquisador: ______________

Rubrica participante: ______________

Manguinhos - Rio de Janeiro – RJ - CEP: 21041-210. O CEP funciona de segunda a sexta-feira, das 9h às 17h.

Se desejar, consulte ainda a Comissão Nacional de Ética em Pesquisa (Conep). A Comissão Nacional de Ética em Pesquisa - CONEP - é uma comissão do Conselho Nacional de Saúde - CNS, criada por meio da Resolução 196/96 com a função de implementar as normas e diretrizes regulamentadoras de pesquisas envolvendo seres humanos, aprovadas pelo Conselho. Tem função consultiva, deliberativa, normativa e educativa, atuando conjuntamente com uma rede de Comitês de Ética em Pesquisa - CEP- organizados nas instituições onde as pesquisas se realizam, em um sistema de acompanhamento das pesquisas realizadas no país, o Sistema CEP/CONEP

Contatos da CONEP:

E-mail: conep@saude.gov.br, Telefone: (61) 3315-2951

Endereço: Ministério da Saúde - Esplanada dos Ministérios Edifício Anexo Bl. G Ala B Sl. 13-B

Cep: 70.058-900 Brasília – DF. Horário de funcionamento: segunda à sexta das 8h às 18h.

________________________________ Gustavo Corrêa Matta (Assinatura do Pesquisador) (Nome do pesquisador)

Declaro que entendi os objetivos de minha participação na pesquisa e concordo em participar.

_______________________________ _________________________________________ (Assinatura do Participante) (Nome do Participante)

______________________, _____ de _________________ de ____________

(Local) (dia) (mês) (ano)

Página 3 de 3

Rubrica pesquisador: ______________

Rubrica participante: ______________
